# A scoping review of the barriers and facilitators in the use of traditional, complementary, and integrative medicine: insights for health policy development

**DOI:** 10.1186/s41043-025-00934-y

**Published:** 2025-06-05

**Authors:** Seyede Maryam Najibi, Yaser Sarikhani, Mahdie Hajimonfarednejad, Majid Nimrouzi, Mohammad Hashem Hashempur

**Affiliations:** 1 Research Center for Traditional Medicine and History of Medicine, Department of Persian Medicine, School of Medicine, Shiraz University of Medical Sciences, Shiraz, Iran; 2 Research Center for Social Determinants of Health, Jahrom University of Medical Sciences, Jahrom, Iran

**Keywords:** Complementary therapies, Integrative medicine, Scoping review, Traditional Persian medicine, Health policy

## Abstract

**Background:**

The historical and cultural importance of traditional, complementary, and integrative medicine (TCIM) is observable in diverse contexts and among different populations. As the use of TCIM continues to grow globally, policymakers need to acknowledge its importance in healthcare services.

**Objectives:**

We conducted a scoping review of quantitative, qualitative, and mixed-method research to identify the factors that promote and hinder the adoption of TCIM.

**Methods:**

This scoping review involved a comprehensive search of online databases from 2000 to February 2024. The review utilized the methodology suggested by Arksey and O’Malley. Qualitative content analysis was employed to synthesize the data.

**Findings:**

From a total of 1403 articles retrieved, 61 full-text articles were chosen for the final analysis. Among these, 47 examined facilitators, 4 addressed barriers, and 10 investigated both barriers and facilitators of using TCIM. Three key themes were recognized concerning barriers to using TCIM services, including “service delivery problems”, “governance challenges”, and “personal barriers”. Six key themes associated with the factors facilitating the use of TCIM services were recognized, which include “financial facilitators”, “health conditions”, “personal determinants”, “perceived benefits”, “social impact”, and “appropriate service delivery”.

**Conclusions:**

Exploring the barriers and facilitators of using TCIM services can provide valuable insights to policymakers, enabling them to develop strategies to overcome existing challenges and enhance the support for the growth of these services. This knowledge is essential for making sure that TCIM services are available to people in a safe, prompt, and high-quality way.

**Supplementary Information:**

The online version contains supplementary material available at 10.1186/s41043-025-00934-y.

## Introduction

Non-communicable diseases, including obesity, represent a significant and growing public health burden worldwide, with complex etiologies and substantial impacts on morbidity and healthcare systems [1]. Given the challenges in managing these conditions, alternative approaches such as traditional, complementary, and integrative medicine (TCIM) offer promising strategies for prevention and treatment by addressing lifestyle and behavioral factors [2].

TCIM encompasses diagnostic, therapeutic, and preventive approaches that complement conventional medicine (CM) by enhancing public health, addressing unmet medical needs, or expanding medicine’s conceptual frameworks [3]. The World Health Organization (WHO) defines traditional medicine (TM) as the comprehensive body of knowledge, skills, and practices rooted in the theories, beliefs, and experiences indigenous to various cultures. This encompasses understandable and unexplainable methods for maintaining health and preventing, diagnosing, enhancing, or treating physical and mental ailments. Additionally, “complementary medicine” and “alternative medicine” refer to a wide range of healthcare practices that do not conform to the conventional or complementary methods, and are completely incorporated into the prevailing healthcare system. In some areas, these terms are occasionally used synonymously with TM [4].

From the Alma-Ata Declaration in 1978 to the Sustainable Development Goals (SDGs) in 2015, global health policy has consistently emphasized healthcare accessibility ‘a trajectory significantly influenced by Alma-Ata’s recognition of TM and its practitioners in primary healthcare. This pivotal shift not only advanced conventional healthcare systems but also fostered the progressive global integration of TM [5]. In May 2014, the World Health Assembly passed Resolution WHA67.18 on TM, urging Member States to formulate and execute policies that integrate traditional and complementary medicine (T&CM) into national healthcare systems, aligned with the WHO Traditional Medicine Strategy 2014–2023 [6].

Contemporary healthcare systems worldwide grapple with persistent challenges, including a growing imbalance between care demand and human and economic resource availability. This complex landscape is further shaped by a fragmented mix of formal and informal care options, disparities in access and cost, and uneven resource distribution [7]. In this context, the utilization of TCIM holds significant historical and cultural importance across different environments and groups, and it can be safe and advantageous when chosen and utilized appropriately [8]. Research indicates that the usage rates of TCIM services differ across various countries. A systematic review conducted in 2022 found that the usage prevalence varied between 24 and 71.3% [9]. Additionally, a survey conducted across 32 countries revealed that the use of TCIM rose from under 10% in Bulgaria, Poland, and Slovenia to more than 50% in China, the Philippines, and Korea [10].

Numerous studies demonstrate the advantages of TCIM—including lifestyle and nutritional modifications—in managing various disorders and diseases, including psychiatric disorders [11], cancer [12–16], non-communicable diseases [17], acute respiratory syndrome [18], and, more recently, COVID-19 [19–21]. The delivery of TCIM treatments and their related products has also led to considerable economic effects. Numerous multinational companies have invested heavily in TCIM, with billions of dollars allocated to it annually across various developed economies. WHO estimates that the yearly worldwide market for TCIM is approximately 83 billion dollars [22]. At the same time, a range of social, political, and economic factors are driving the growing use of TCIM. Factors include the patient’s willingness to take an active part in health decisions [23], the low frequency of side effects [24], as well as accessibility and cultural standards [25, 26]. Additionally, barriers to the use of TM services are emphasized, including the lack of definitive scientific evidence supporting the efficacy of TCIM [27], the high prices of herbal treatments [28], and the lack of regulations governing TCIM products and practitioners [29].

Despite the recent increase in the application of TCIM interventions and their recognized benefits for enhancing both individual and community health, a thorough review of existing evidence reveals that a comprehensive analysis focused on identifying the barriers and facilitators to the effective use of these services has yet to be conducted. An effective strategy for stimulating the rightful use of TCIM requires a comprehensive understanding of the variables that either facilitate or hinder its adoption. In this context, limitations in the diversity of study populations, diversity in conceptual models, and the dispersion of findings have made the achievement of a singular and reliable comprehension elusive. Consequently, it is perceived as necessary to conduct a scoping review that will clarify, categorize, and analyze these variables. Because of the extensive range of subject matter and heterogeneity of the available literature, a scoping review approach was selected as an appropriate method for mapping the core concepts and identifying research gaps [30]. Therefore, we aimed to identify the factors that promote and hinder the use of TCIM services worldwide. The findings of this study can provide a basis for establishing evidence-based interventions, refining public health policy, and guiding future research on the subject of TCIM.

## Methods

This scoping review follows the checklist for systematic reviews and meta-analyses Extension for Scoping Reviews (PRISMA-ScR) [31]. This approach is useful for collecting a wide range of literature within a specific area of study. It enables the incorporation of literature with various designs and sample types. Unlike systematic reviews, quality assessment of individual studies is typically not conducted [32].

### Study design

This research employed a scoping review method to pinpoint the obstacles and enablers associated with TCIM utilization. Due to the growing worldwide use of TCIM and its related health and economic benefits, a scoping review was conducted to gain a thorough understanding of key concepts, including the obstacles and factors influencing the use of TCIM. This type of research allows for the recognition of important elements affecting a topic, helps in developing a thorough summary of pertinent evidence, and highlights areas where current research is lacking in the given field [33]. The method proposed by Arksey and O’Malley was utilized to perform a scoping review. This method consists of five stages: 1- determining the research question, 2- searching and retrieving studies, 3- selecting related studies, 4- data charting, and 5- collating and summarizing results [34].

### Determining the research question

This research was conducted to address the inquiry: "What are the facilitators and barriers associated with the utilization of TCIM services?".

### Searching and retrieving studies

We identified pertinent keywords related to the research question using the MeSH and Emtree databases. A suitable search strategy was developed for each database, which included PubMed, Scopus, Web of Science, Science Direct, and Google Scholar. Articles released from 2000 to February 2024 were chosen for retrieval. The search strategy for the scoping review is outlined in Table 1.

**Table 1:** The search strategy of the study

Databases	PubMed, Scopus, Web of Science, ScienceDirect, Google Scholar
Limit	Language: Full text in English Time: 2000 to February 2024
Search Strategy	**#1 AND #2 AND #3**
#1	Barriers [Title/Abstract] **OR** Facilit*[Title/Abstract] **OR** Inhibit*[Title/Abstract] **OR** “Factors affect” [Title/Abstract] **OR** “Affecting factor” [Title/Abstract] **OR** “Effecting factor” [Title/Abstract] **OR** “Factors effect” [Title/Abstract] **OR** Challenge **OR** Predict* [Title/Abstract] **OR** Obstacle [Title/Abstract]
#2	((“Complementary Therapies” [MeSH Terms] **OR** “Medicine, Traditional” [MeSH Terms] **OR** “Complementary medicine” [Title/Abstract] **OR** “Alternative Medicine” [Title/Abstract] **OR** “Alternative therapy” [Title/Abstract] **OR** “Traditional medicine” [Title/Abstract] **OR** “Herbal medicine” [Title/Abstract]))
#3	Use [Title/Abstract] **OR** Utilization [Title/Abstract] **OR** Usage [Title/Abstract] **OR** Access [Title/Abstract] **OR** Accessibility [Title/Abstract] **OR** Using [Title/Abstract]

**PubMed**: (((((((use[Title]) OR (Utilization[Title])) OR (usage[Title])) OR (Access[Title])) OR (Accessibility[Title])) OR (using[Title])) AND ((((((((((Barriers[Title]) OR (Facilit*[Title])) OR (Inhibit*[Title])) OR ("Factors affect"[Title])) OR ("Affecting factor"[Title])) OR ("Effecting factor"[Title])) OR ("Factors effect"[Title])) OR (Challenge[Title])) OR (Predict*[Title])) OR (Obstacle[Title]))) AND ((((((("Complementary Therapies"[MeSH Terms]) OR ("Medicine, Traditional"[MeSH Terms])) OR ("Complementary medicine"[Title/Abstract])) OR ("Alternative Medicine"[Title/Abstract])) OR ("Alternative therapy"[Title/Abstract])) OR ("Traditional medicine"[Title/Abstract])) OR ("Herbal medicine"[Title/Abstract]))

### Selecting related studies

The search results for relevant articles were inputted into Endnote X21 software (Thomson Reuters, New York, NY), where duplicates were removed. The remaining studies were then reviewed by title and abstract to assess their relevance to our objectives. The criteria for including studies were based on three main aspect of the scoping review studies, including population, concepts, and context (PCC) which were applied during both the evaluation and inclusion stages. This scoping review examines research involving all populations, encompassing different disease categories as well as healthy individuals, and does not impose any age limitations (population). We included all quantitative and qualitative research that discussed the obstacles and enablers related to the use of TCIM. TCIM is defined differently across different nations. To ensure we captured all relevant data, we did not impose any limitations on the types of TCIM modalities. This review encompasses research on herbal medicine, different manual techniques like massage, and various holistic approaches, including traditional Iranian medicine, traditional Chinese medicine, and Indian medicine (concept). Additionally, the review included research from different countries around the world (context). The exclusion criteria included: protocol studies; letters to the editor, abstracts, and editorials; studies lacking full text in English; articles without full text; review studies; and studies that did not align with the research objective.

**Fig. 1:**
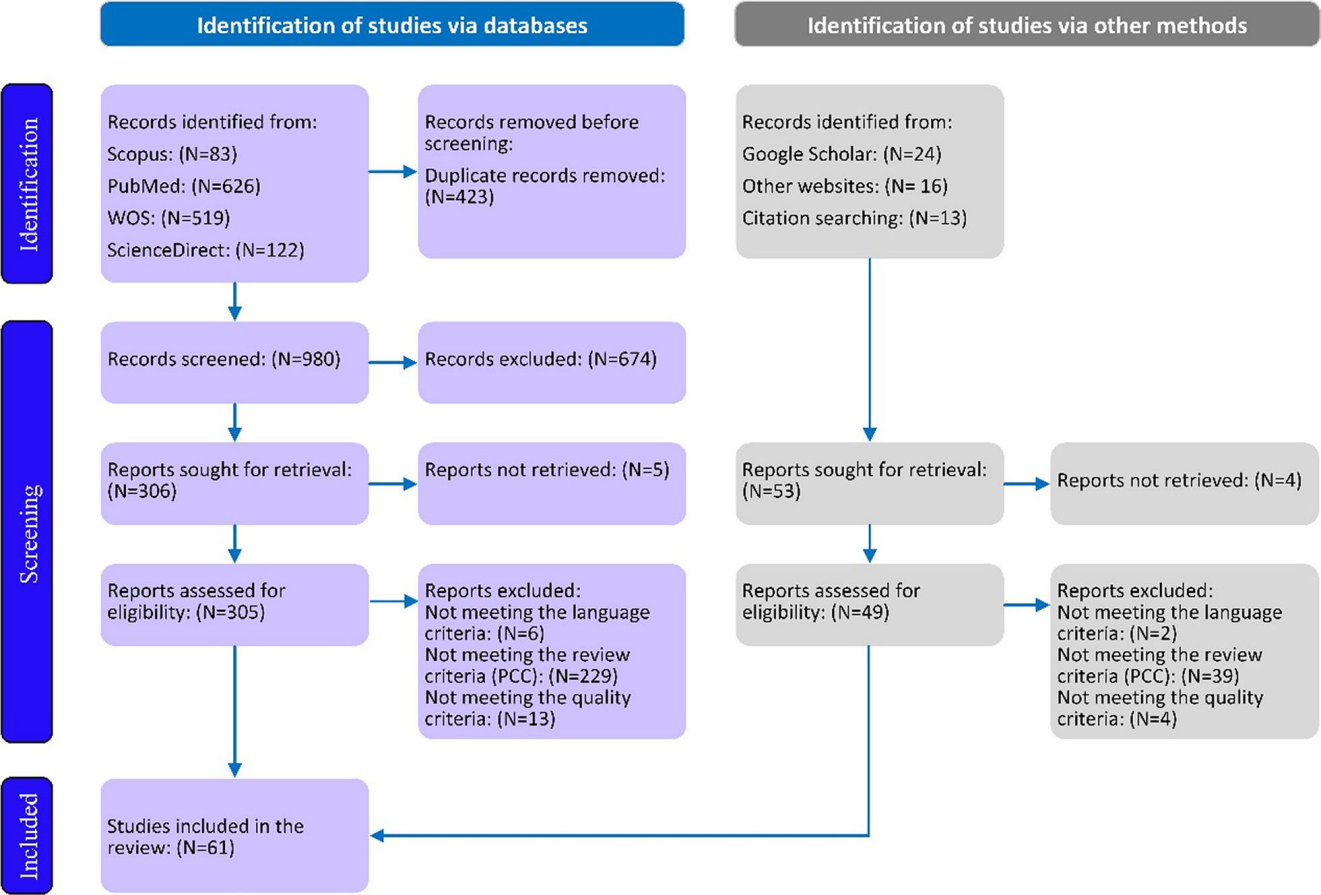
PRISMA flow diagram of the study

To find pertinent studies, two reviewers (SMN and MH) carefully analyzed the full texts of the selected articles. Any inconsistencies in the evaluation process were addressed by a third reviewer (YS). The process of selecting articles is illustrated in a PRISMA flow diagram shown in Fig. 1.

### Data charting

A data-charting form was created to aid in the extraction of data. Three researchers worked together in an iterative manner to carry out the data-charting process, which allowed for ongoing extraction and updates of the information in the form. The items in this form consist of: (1) the name of the first author, (2) the year of publication, (3) the country where the study was conducted, (4) the title of the study, (5) the type of TCIM, (6) the study design, and (7) the facilitators and obstacles to the use of TCIM (Supplementary Table 1).

### Collating and summarizing results

We employed a six-step method of thematic content analysis, as outlined by Graneheim and Lundman, to synthesize the data. This process includes becoming acquainted with the data, conducting initial coding, identifying themes, reviewing the themes, defining them, and finally reporting the results [34, 35]. Two researchers (SMN and YS) performed separate data analyses and then compared their results to ensure consistency and address any differences. In the first stage of thematic analysis, the researchers immersed themselves in the data by reading the full text of the articles several times. They created initial codes based on the goals of the study. They conducted an interpretive analysis of the initial codes to identify the main themes and their related sub-themes. Subsequently, the themes were evaluated in a collaborative discussion, where the research team adjusted, merged, divided, or removed main themes as needed. Ultimately, the research team identified and categorized the themes and sub-themes based on their relevancy. We created a thematic network of the evidence presented as a conceptual map to provide a comprehensive understanding of the factors that promote and hinder TCIM usage.

## Results

### Characteristics of included studies

Our search resulted in the extraction of 1403 articles. Following a three-step review process, we chose 61 full-text articles for the final analysis. Out of the chosen articles, 47 (77.04%) examined the factors that promote the use of TCIM, 4 (6.4%) addressed obstacles, and 10 (16.1%) investigated both barriers and facilitators. The majority of the articles were released between 2011 and 2015. Over 83.6% (N = 51) of the research was carried out in high and upper-middle-income nations, with the USA and then Australia leading in the number of articles, having 14 (22.9%) and 9 (14.5%) studies, respectively. Ghana had the highest number of articles (N = 5) among low-and lower-middle-income countries (LLMICs). The majority of the studies employed a quantitative design. Additionally, 77.04% of the studies concentrated on CAM. The characteristics of the articles included in the thematic analysis are shown in Table 2.

**Table 2:** Characteristics of the articles selected for the final analysis

Characteristics	Frequency and (%) of included articles
Publication year	
2001–2005	7 (11.2%)
2006–2010	12 (19.3%)
2011–2015	19 (31.1%)
2016–2020	13 (20.9%)
2021 ≤	10 (16.1%)
Study Location	
Low- and lower-middle-income	10 (16.1%)
High and upper-middle income	51 (83.6%)
Study type	
Quantitative	52 (85.2%)
Qualitative	6 (9.6%)
Mixed methods	3 (4.8%)
Type of TCIM	
CAM	47 (77.04%)
Herbal medicine	8 (13.1%)
TM	4 (6.5%)
TCIM	2 (3.2%)

### Thematic analysis and reporting

**Fig. 2:**
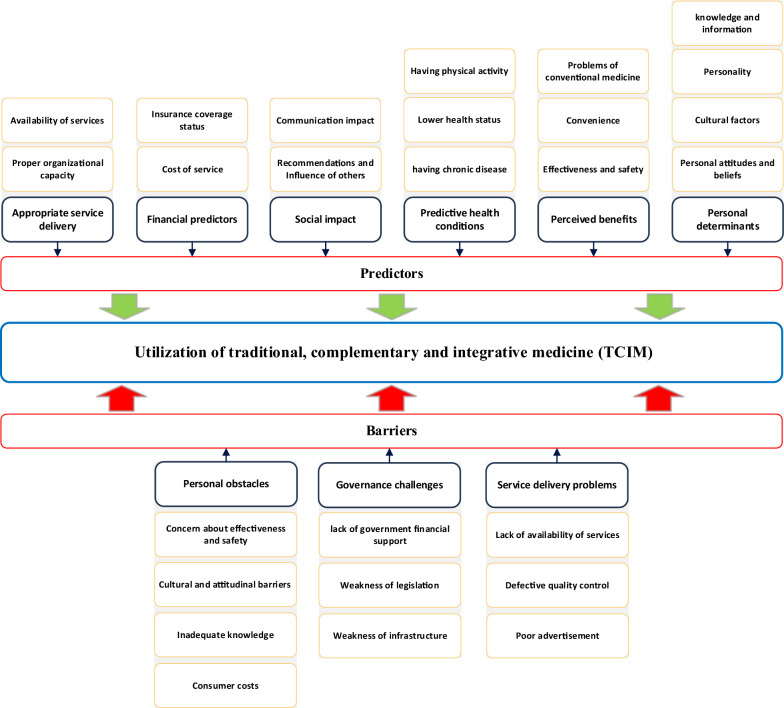
Conceptual framework of evidence on key barriers and facilitators of TCIM utilization

The thematic analysis revealed three main themes and ten sub-themes related to the barriers to using TCIM services. Additionally, six main themes and sixteen sub-themes were identified as facilitators for utilizing TCIM services, as shown in Table 4. The conceptual framework derived from the thematic analysis is illustrated in Fig. 2.

### Barriers to the utilization of TCIM services

The key obstacles to the use of TCIM include insufficient scientific evidence [36–39], a shortage of qualified professionals [36, 37, 40], limited service availability [41, 42], difficulties in accessing TCIM practitioners and services [43, 44], inadequate advertisement [42, 45], lack of government financing for TCIM [36, 40], legal concerns surrounding TCIM practices [36, 40], and a general lack of awareness about CAM [37, 44]. The results are presented in Table 3.

**Table 3:** Themes and sub-themes related to the barriers to using TCIM services

Themes	Sub-themes	Categories
Service delivery problems	Lack of availability of services	Lack of availability of services [41, 42] Shortage of professionals [36, 37, 40] Limitation of accessing TCIM practitioners and services [43, 44] Transportation problem [42] Long waiting time for treatment [37]
	Defective quality control	Poor quality control of herbal medicine [41] Risk of developing drug-resistant [46] Lack of scientific evidence [36–39]
	Poor advertisement	Poor advertisement [42, 45] Poor knowledge of vendors [47]
Governance challenges	lack of government financial support	Lack of government financial support for TCIM [36, 40]
	Weakness of legislation	Concerns about the legal issues of the use of TCIM [36, 40] Absence of formal regulation on herbal medicines [41] Lack of effective communication between practitioners and health authorities [41]
	Weakness of infrastructure	Insufficient infrastructure to sustain the service [45] Poor vending environment [47] Difficulty in retaining a rural TCIM workforce [43]
Personal barriers	Consumer costs	Out-of-pocket payments [48] High cost of herbal products [47] Cost of trip [43]
	Inadequate knowledge	Lack of previous experience with the use of CAM [38] Lack of knowledge about CAM [37, 44] Limited information on their adverse events [41] Lack of awareness of the availability of TCIM services [45]
	Cultural and attitudinal barriers	Religion beliefs [49] Cultural factors [49] Feeling obligated to conventional medical practitioners [49] Negative perceptions and attitudes about herbal medicine [47] Lack of interest in using CAM [49] Perceived social pressure [50]
	Concern about effectiveness and safety	Inconsistent effectiveness of some herbal product [47] Low effect of herbal treatment [45] Concern about side effects [51] Harmful drug interactions [51]

### Facilitators to the utilization of TCIM services

The studies that were analyzed highlighted factors that promote the use of TCIM services (Table 4). The most common factors that facilitate TCIM include experiencing chronic and long-term health issues [52–62], having a lower health status [63–68], affordable cost [41, 68–71], spirituality [47, 67, 72–74], religious belief [49, 54, 63, 72, 75], TCIM services covered by the national health insurance plan [45, 51, 52, 54], as well as personal characteristics and subjective norms [50, 59, 70, 76].

**Table 4:** Themes and sub-themes related to the facilitators of using TCIM services

Themes	Sub-themes	Categories
Financial facilitators	Cost of service	Affordable cost [41, 68–71] No cost [46]
	Insurance coverage status	TCIM services covered by the National Health Insurance scheme [45, 52, 54, 51] Lack of basic insurance [53, 73]
Predictive health conditions	having chronic disease	Suffering from chronic and longstanding health problems [52–62]
	Lower health status	Lower health status [63–68]
	Having physical activity	More physical and leisure activities [55, 57, 77]
Personal determinants	Personal attitudes and beliefs	Personal preference for herbal medicine [47, 51] Personal behavioral beliefs [76] Personal attributes and subjective norms [59, 70, 76, 50] Beliefs related to control and participation [62, 50] Perceptions of illness [78] Past behavior about CAM use [50] Positive experience with the use of CAM [59, 74] Positive attitude of the health workers [46] Attitude towards CAM practitioners [79] Positive attitude toward effectiveness, safety, and cost of CAM [74, 80, 81] Health Care Encounter Outcome Expectancies [79] Positive personal values outcome expectancies [79]
	Cultural factors	Cultural characteristics [42, 71, 82] Spirituality [66, 47, 72–74] Religiousness [54, 63, 72, 73, 75]
	Personality	Special personality characteristics [42, 83] Desire to take responsibility for own health [43, 66] Self-efficacy feeling [50]
	knowledge and information	More knowledge about TCIM [42, 45] Awareness of the TCIM services [37]
Perceived benefits	Effectiveness and safety	Lower side effects and higher safety [70, 84] Treatment success stories [42]
	Convenience	More convenient to use HM than other medicines [37, 70]
	Problems of Conventional Medicine	Perceived ineffectiveness of CM and integration of spirituality in herbal medicine [85] Concern about the safety of CM [41, 86] Dissatisfaction with the CM services [61, 48, 81] Experienced delays in CM care and unmet needs [61, 49, 48] Limitation of access to conventional health services in rural areas [43]
Social impact	Recommendations and Influence of Others	Recommendation from family [43] Recommendations from other people [52] Recommendation by health care professionals [39] Prescribe by non-medical practitioners [74] Better understanding of pharmacists of CAM [85] Good effective behavior of the traditional medical practitioner [59] Increasing referrals by general practitioners [65]
	Communication impact	Impact of social contacts [42] Media [42] Effect of social networks [43]
Appropriate service delivery	Proper organizational capacity	Political support [45] Public support [45] Funding [45] Organizational capacity [45] Programmed evaluation [45] Partnership and planning [45]
	Availability of services	Easy access to practitioners and services [43, 45]

Figure 2 illustrates the conceptual framework derived from the thematic analysis findings.

## Discussion

In this research, we examined various studies on the barriers and facilitators of TCIM utilization. We analyzed 61 studies, the majority of which were conducted in high and upper-middle-income countries.

### Barriers to the use of TCIM services

The findings showed that, among the studies reviewed, the most common obstacle to the utilization of TCIM services was the lack of scientific evidence. Similarly, Leach and Veziari (2022) identified limited access to bibliographic databases and insufficient time for practitioners to engage with evidence-based resources [87]. Healthcare professionals frequently highlight the absence of evidence supporting TCIM [88]. While there has been a significant increase in evidence related to TCIM in the last two decades, a considerable amount of additional evidence is still required to enhance our understanding of TCIM. Evidence maps are essential for promoting evidence-based practice, guiding decision-making for managers, healthcare providers, and patients, and identifying research priorities to address existing knowledge gaps [89]. Addressing the lack of scientific evidence requires establishment of a Virtual Health Library (VHL) focused on TCIM [89]. This initiative should create collaborative international research networks specifically aimed at TCIM, encouraging multi-country, cross-cultural studies and the sharing of resources [87]. Additionally, the WHO suggests developing training programs to enhance skills in critical appraisal, research literacy, and evidence generation among TCIM providers [90].

In addition to systemic issues, a main barrier identified in this review is personal barriers. These barriers include consumer costs, insufficient knowledge, cultural and attitudinal barriers, and concerns about effectiveness and safety. Each of these factors underscores the deeper complexities within the patient decision-making process when considering TCIM. Consistent with our study, a review noted that personal barriers—including financial burden, lack of consumer knowledge, cultural hesitation, and safety concerns—restrict the use of TCIM services [91]. The barriers to the TCIM use are complex and multifaceted, encompassing both systemic issues and individual challenges. Structural problems, such as the lack of high-quality scientific evidence, intersect with personal factors. These barriers are interconnected, reinforcing each other in intricate ways. The absence of evidence-based research in TCIM contributes to uncertainty among consumers and hesitancy among professionals [92].

Addressing these challenges requires a comprehensive strategy that reforms the evidence-generation process, enhances educational outreach, ensures financial accessibility, and promotes culturally sensitive engagement with TCIM. Without these integrated efforts, the potential contributions of TCIM to global health systems will remain limited, and opportunities for more patient-centered and diverse models of healthcare delivery will continue to be underutilized.

According to the results of this study, in LLMICs, Lack of availability of services expressed as obstacle to the use of TCIM. The ability to access health services greatly influences the selection of treatment options. Nevertheless, access is not consistent for everyone [26, 93]. As global initiatives continue to promote fair access to quality healthcare, successfully combining TCIM with conventional treatments could be a key focus for enhancing accessibility [26].

Many LLMICs struggle with inadequate infrastructure to provide TCIM services. There is often a shortage of dedicated facilities, necessary equipment, and trained personnel needed to deliver these services effectively [94]. The integration of TCIM into mainstream health facilities is frequently incomplete or unsustainable due to insufficient funding, a lack of organizational capacity, and inadequate infrastructure. Additionally, many countries do not formally recognize, regulate, or integrate TCIM within their national health systems. This results in fragmented service delivery, poor quality control, and limited official support for expanding access to TCIM services [95].

### Facilitators to the use of TCIM services

Predictive health conditions play a crucial role in motivating individuals to utilize TCIM. Suffering from chronic and long-standing health problems, along with a lower health status, has been identified as a facilitator in 17 different studies. Individuals with chronic health conditions often experience persistent symptoms and treatment side effects that CM may not completely alleviate. The belief that TCIM can enhance overall health and provide symptom relief encourages patients to incorporate these methods into their treatment plans [6, 96]. Many patients have reported that TCIM methods help them manage e their symptoms more effectively and reduce their reliance on CM [96]. Furthermore, the WHO acknowledges the long-standing role of TM in CM and its ongoing ability to address chronic health problems [97].

The results of this review indicated that some studies have identified cost as a barrier, while others consider it as a supportive factor. The expenses associated with TCIM services vary significantly from one country to another, influenced by factors such as local healthcare systems, resourcesavailability, and specific practices used. The pricing of TCIM services is affected by a variety of interconnected factors, including workforce availability, infrastructure, regulations, demand trends, cultural acceptance, and the economic situation in the area. Understanding these components is crucial for creating successful approaches to improve the accessibility and sustainability of TCIM across different healthcare systems globally [98, 99]. For instance, the issue of herbal medicines in LLMICs dealing with economic challenges is a major concern, as it increases the risk of users purchasing products from unreliable sources at lower prices [100]. In China, traditional medicine practices are cost-effective for health systems and insurance frameworks, highlighting the financial advantages of incorporating TCIM into conventional healthcare [101, 102].

Furthermore, the study’s findings indicated in numerous studies expressed personal attitudes, beliefs and culture factors as common facilitators of TCIM use. This is consistent with previous studies. These factors interact to create environments where TCIM is not only accepted but often preferred, especially when conventional healthcare is perceived as inadequate, inaccessible, or culturally misaligned [26, 96, 103]. In different cultures, religious beliefs and spirituality play a significant role in shaping individuals’ preferences and approaches to TCIM [104]. The variety of TCIM practices found in different cultures and areas emphasizes the importance of adaptable policies that acknowledge and integrate these diverse methods. This is important because TCIM encompasses various therapies and techniques that are strongly influenced by cultural and historical backgrounds [105]. Understanding these facilitators is crucial for healthcare providers seeking to engage with patients in culturally sensitive and effective ways.

### Policy recommendations

Health systems are encouraged to improve the accessibility of services by integrating TCIM into standard healthcare systems and increasing service locations, especially in underserved regions. Quality control can be enhanced by implementing standardized regulations and certifications for TCIM products and practitioners to guarantee safety and effectiveness. Public awareness campaigns and advertisements must be improved to inform the community about the available TCIM services and their advantages. Governments can improve their financial assistance by allocating dedicated funds for TCIM research, infrastructure, and service provision, which includes reimbursement programs for TCIM treatments. Finally, addressing cultural and attitudinal challenges requires the engagement of community leaders and the modification of TCIM services to reflect cultural beliefs and preferences.

### Strengths and limitations of the study

This study was designed as a scoping review to identify the barriers and facilitators to the use of TCAM services globally. However, several limitations should be considered when interpreting the results. First, this study did not assess the methodological quality of the included articles, which is a characteristic feature of scoping reviews. Second, although qualitative content analysis is suggested as a suitable approach for data synthesis in scoping reviews, incorporating findings from various data collection methods as a form of triangulation can enhance the results. Third, cultural diversity, variations in health systems, and the scope of TCIM services across different countries limit the direct comparison of results. In light of these limitations, it is recommended that future research focus on in-depth qualitative studies to gain a more comprehensive understanding of the cultural and social contexts surrounding the barriers and facilitators to the use of TCAM. Furthermore, conducting international comparative studies could help clarify the role of health systems and national policies in the utilization of these services. Future studies should also examine the diverse perspectives of patients, healthcare providers, and policymakers separately. Moreover, considering that a significant number of studies in this field have been carried out in high-income nations, it is essential to interpret the findings with caution and concerning the specific country context.

## Conclusion

This scoping review employed the Arksey and O’Malley approach to identify and categorize the barriers and facilitators associated with the use of TCIM services. Three main themes that explain the obstacles to using TCIM including service delivery problems, governance challenges, and personal barriers. Conversely, the facilitators of TCIM use were organized into six key themes.

A comprehensive understanding of these factors is essential for developing effective policies aimed at promoting the integration of TCIM into health systems. The findings of this study can inform the development of interventions that mitigate barriers and strengthen facilitators, thereby enhancing access to and acceptance of TCIM within the framework of mainstream health care. Such an approach can improve responsiveness to patients’ needs and preferences, promote patient-centered care, and ultimately lead to better health outcomes. Given the dearth of investigations into the barriers to TCIM utilization, this issue should be considered an area for further investigation.

## Supplementary Information

Additional file 1.

## Data Availability

The data that support the findings of this study are available from the corresponding author upon reasonable request.
