# Supplementary material for: A scoping review of the barriers and facilitators in the use of traditional, complementary, and integrative medicine: insights for health policy development
Source: J Health Popul Nutr. 2025 Jun 5;44:188. doi: 10.1186/s41043-025-00934-y (PMC12139373; doi:10.1186/s41043-025-00934-y)
Supplement: Supplementary file 1 — Additional file 1. [file 41043_2025_934_MOESM1_ESM.docx]

**Supplementary Table 1: Data Charting Form**

|  | **Author/ year** | **Title** | **Type of TCIM** | **Journal** | **location** | **Design** | **Key Findings** |
| --- | --- | --- | --- | --- | --- | --- | --- |
|  | Afriyie et al., 2023 | Predictors of herbal medicine use in Ashanti Region of Ghana | Herbal medicine | Advances in Integrative Medicine | Ghana | Cross-sectional | **Predictors**:  1-Living in a district  2- Having the opinion that herbal medicines work very well  3-use herbal medicine with ease |
|  | Agyei-Baffour et al., 2017 | Integrating herbal medicine into mainstream healthcare in Ghana: clients' acceptability, perceptions and disclosure of use | Herbal medicine | BMC Complementary and Alternative Medicine | Ghana | Cross-sectional | **Predictors**:  1-Being effective  2- Easy to access  3- Being comparatively cheaper  4-Cost of HM was affordable  6- If herbal formulations are proven to be an effective treatment option |
|  | Al-Windi et al., 2004 | Determinants of complementary alternative medicine (CAM) use | CAM | Complementary therapies in medicine | Stockholm, Sweden | Cross-sectional | **Predictors**:  1- Patients with chronic disease and physical activity |
|  | Al-Windi et al., 2004 | Predictors of herbal medicine use in a Swedish health practice | Herbal medicine | pharmacoepidemiology and drug safety | Stockholm, Sweden | Cross-sectional | **Predictors**:  1- Patients born in Nordic countries or Europe  2- Patients with chronic disease |
|  | Aziato et al., 2016 | Facilitators and barriers of herbal medicine use in Accra, Ghana: an inductive exploratory study | Herbal medicine | BMC Complement Altern Med | Ghana | Inductive exploratory qualitative approach | **Predictors**:  1- Convincing information  2-Effectiveness of herbal medicine  3-Personal preference for herbal medicine  4-Perceived ineffectiveness of Western medicine and integration of spirituality in herbal medicine.  **Barriers**:  1-Negative perceptions and attitudes about herbal medicine  2-Poor vending environment  3-Poor knowledge of vendors  4-High cost of herbal products at credible herbal clinics  5- Inconsistent effectiveness of some herbal products |
|  | Babar et al., 2012 | Perceptions and self-use of Complementary and Alternative Medicine (CAM) among Malaysian dental students | CAM | European Journal of Integrative Medicine | Malaysia | Cross-sectional | **Predictors**:  1-Effective  2- Not harmful  **Barriers**:  1-Lack of scientific evidence  2-Lack of trained professionals  3-Lack of government subsidies  4-Concern about legal issues  5-Too time consuming |
|  | Barbadoro et al., 2011 | Complementary and Alternative Medicine (CAM) among adults in Italy: Use and related satisfaction | CAM | European Journal of Integrative Medicine | Italy | Cross-sectional | **Predictors**:  1-living in Northern Italy  2-People with chronic diseases |
|  | Ben Natan et al., 2016 | Factors Related to the Intention of Israelis to Use Complementary and Alternative Medicine | CAM | J Holist Nurs | Israel | A quantitative correlational study | **Predictors**:  1-Behavioral beliefs  2-The family  3-Behavioral attitudes  4-Normative beliefs |
|  | Bishop et al., 2006 | Why do people use different forms of complementary medicine? Multivariate associations between treatment and illness beliefs and complementary medicine use | CAM | Psychology & Health | United Kingdom | A correlational web-based questionnaire study | **Predictors**:  1-Holistic health beliefs  2-Beliefs in emotions as a cause of illness |
|  | Bohm et al., 2012 | Examining the influence of insurance and moral hazard on the utilization of practitioner-based complementary and alternative medicine | CAM | Focus on Alternative and Complementary Therapies | United States | Cross-sectional | **Predictors**:  1-Health insurance |
|  | Buchwald et al., 2000 | Use of traditional health practices among Native Americans in a primary care setting | Traditional health practices | Med Care | United States | Cross-sectional | **Predictors**:  1-Visiting friends/relatives  2-Living the Native way of life  3- Having a physical injury inflicted by a family member |
|  | Čavojová et al., 2020 | The role of scientific reasoning and religious beliefs in use of complementary and alternative medicine | CAM | Journal of Public Health | Slovakia | Cross-sectional | **Predictors**:  1-Scientific reasoning  2-Religious faith |
|  | Cernasev et al., 2020 | Use of National Consumer Survey Data to Explore Perceptions of Complementary and Alternative Medicine | CAM | Innov Pharm | United States | Mix method | **Predictors**:  1-Physicians can play a role in creating a balance between prescription use and CAM  2-More effective  3-Better understanding of pharmacists of CAM |
|  | Cizmesija et al., 2008 | Use of complementary and alternative medicine among the patients in primary health care | CAM | Acta Med Croatica | Zagreb, Croatia | Cross-sectional | **Predictors**:  1-Musculoskeletal diseases  2-Gastrointestinal diseases  3- For prevention  4-They believe CAM will help them  5- Failed conventional medicine to alleviate their complaints  5-Afraid side effects of drugs |
|  | Ellison et al., 2012 | Spiritual and religious identities predict the use of complementary and alternative medicine among US adults | CAM | Preventive medicine | United States | Cross-sectional | **Predictors**:  1-Spirituality and religiousness |
|  | Fjær et al., 2020 | The use of complementary and alternative medicine (CAM) in Europe | CAM | BMC Complement Med Ther | 21 European countries | Cross-sectional | **Predictors**:  1-visits to health care practitioners  2-Less financial strain  3-Unmet need  4-Dissatisfaction with health services  5-Having a longstanding health problem  6-Health expenditures per capita  7-Physician density  8-greater resources  **Barriers**:  1-Out-of-pocket payments  2-Gatekeeping |
|  | Franic and Kleyman., 2012 | A qualitative study of herbal medicine use in the Ukraine: implications for US pharmacy practice | Herbal medicine | J Pharm Pract | Ukraine | Qualitative study | **Predictors**:  1-Herbal medications are less harmful  2-Being safer with no long-term side effects herbal medications  3-Cost |
|  | Fravel et al., 2023 | Dietary supplement and complementary and alternative medicine use among older adults in Australia and the United States | CAM | J Am Geriatr Soc | Australia and the United States | Cross-sectional | **Predictors**:  1-Country of residence  2-Polypharmacy (hose taking five or more prescription medications)  3-Living situation |
|  | Furnham and Lovett., 2001 | Predicting the use of complementary medicine: A test of the theories of reasoned action and planned behavior | CAM | Journal of Applied Social Psychology | United Kingdom | Cross-sectional | **Predictors**:  1-Attitudes  2-subjective norms  3-perceived control  4-perceived social pressure  5-self-efficacy  6-past behavior |
|  | Ganasegeran et al., 2014 | Psycho-socioeconomic factors affecting complementary and alternative medicine use among selected rural communities in Malaysia: a cross-sectional study | CAM | PLoS One | Malaysia | Cross-sectional | **Predictors**:  1-Monthly household income  2-Positive attitude towards CAM  3-Higher education level  4-Unemployed occupants  5-Occupants with positive holistic health beliefs |
|  | Gupchup et al., 2006 | Relationships between Hispanic ethnicity and attitudes and beliefs toward herbal medicine use among older adults | Herbal medicine | Res Social Adm Pharm | Hispanic | Cross-sectional | **Predictors**:  1-Intention and attitude toward using herbal medicines  ethnicities  2-Herbal medicines are cheaper  3- Have fewer side effects  4-Work better  5- More convenient to use than other medicines. |
|  | Gyasi et al., 2015 | Predictors of Traditional Medicines Utilization in the Ghanaian Health Care Practice: Interrogating the Ashanti Situation | Traditional Medicines | J Community Health | Ghana | Cross-sectional | **Predictors**:  1-Low-income levels  2-Perceiving TRM as effective and safe  3-Good affective behavior of traditional medical practitioner  4-Having chronic ill-health  5-People’s experience  6- Personal attributes  7-Health beliefs  8-Attitude of TMP to clients and medical history |
|  | Harnett et al., 2023 | Prevalence and Characteristics of Australians Complementary Medicine Product Use, and Concurrent Use with Prescription and Over-the-Counter Medications-A Cross Sectional Study | CAM | Nutrients | Australia | Cross-Sectional | **Predictors**:  1- Having a chronic illness |
|  | Harnett et al., 2019 | Use of complementary medicine products: a nationally representative cross-sectional survey of 2019 Australian adults | CAM | BMJ Open | Australia | Cross-sectional | **Predictors**:  1-Employment status  2-Chronic health condition  3-Those with no private health insurance |
|  | Hasan et al., 2011 | Understanding, perceptions and self-use of complementary and alternative medicine (CAM) among Malaysian pharmacy students | CAM | BMC Complement Altern Med | Malaysia | Cross-sectional | **Barriers**:  1-Lack of scientific evidence  2-Lack of trained professionals in CAM  3-Lack of government financial support for CAM  4-Concerned about the legal issues of the use of CAM  5-Too time consuming to use CAM |
|  | Hasnie et al., 2023 | The Relationships Between Acculturation and Complementary and Alternative Medicine Beliefs and Use Among Asian Americans. | CAM | Asian American Journal of Psychology | United States | Cross-sectional | **Predictors**:  1-Higher acculturation levels (strong affiliations with either heritage culture or host culture) |
|  | Hilal and Hilal., 2017 | Knowledge, attitude, and utilization of herbal medicines by physicians in the Kingdom of Bahrain: A cross-sectional study | Herbal medicines | [Journal of the Association of Arab Universities for Basic and Applied Sciences](https://www.sciencedirect.com/journal/journal-of-the-association-of-arab-universities-for-basic-and-applied-sciences) | Bahrain | Cross-sectional | **Predictors**:  1-Availability of sufficient knowledge  2-Low cost of herbal  3-Medicines  4-Deficits and unfulfilled goals of conventional medicines  5-Side effects and adverse events of conventional medicines  **Barriers**:  1-Poor quality control  2-limited information on their adverse events  3-Absence of formal regulation on herbal medicines  4-Not effective communication with health authorities regarding herbal medicines  6-Fear of professional liability concerning utilizing herbal medicines  7-Lack of availability of herbal medicines. |
|  | Hmwe et al., 2020 | Older people's perspectives on use of complementary and alternative medicine and acupressure: A qualitative study | CAM | Complement Ther Clin Pract | Australia | Qualitative study | **Barriers**:  1-Feeling obligated to conventional medical practitioners  2-Religion beliefs  3-Cultural factors  4-Lack of interest in using CAM. |
|  | Hsiao et al., 2006 | Complementary and alternative medicine use among Asian-American subgroups: prevalence, Predictors, and lack of relationship to acculturation and access to conventional health care | CAM | J Altern Complement Med | California | Cross-sectional | **Predictors**:  1-Who were uninsured or experienced delays in receiving conventional medical care  2-Religiosity  3-More spiritual  4-More education |
|  | Jong et al., 2015 | A cross-sectional study on Swedish licensed nurses' use, practice, perception and knowledge about complementary and alternative medicine | CAM | Scand J Caring Sci | Netherlands | Cross-sectional | **Barriers**:  1-Lack of knowledge  2-Lack of scientific evidence  3-Lack of experience |
|  | Kenu et al., 2021 | Factors that promote and sustain the use of traditional, complementary and integrative medicine services at LEKMA hospital, Ghana, 2017: an observational study | TCIM | BMC Complement Med Ther | Ghana | Mixed methods | **Predictors**:  1-More knowledge (education and awareness creation)  2-Location of TCIM services  3-Perceived effectiveness  4-Availability  5-Funding  6-Organizational capacity  7-Programmed evaluation  8-Political support  9-Partnership and planning  10-Political support from local authorities  11-Public support community stakeholder support  12-Ensuring that TCIM services are integrated into the national health insurance scheme.  **Barriers**:  1-Not gotten sustained funding from the government  2-Infrastructure to sustain the service is not enough  3-Not even aware this service is available  4-Low effect of herbal treatment  5-Lack of advertisement |
|  | Kim et al., 2019 | The Use of Complementary and Alternative Medicine among Korean Young Adult Members of Fitness Centers | CAM | Evid-Based Complement Alternat Med | South Korea | Cross-sectional | **Predictors**:  1-Employed  2-Having a religion  3-Presence of health problems |
|  | Leach., 2021 | Determinants of Complementary Medicine Service Utilization in a Regional South Australian Population: A Cross-Sectional Study | CAM | J Rural Health | Australia | Cross-Sectional | **Predictors**:  1-Employed  2-Income  3-Negative experiences with conventional healthcare services  4-Supportive attitude  5-Living in a remote location  6-Having dependent children |
|  | Lee et al., 2010 | How does acculturation affect the use of complementary and alternative medicine providers among Mexican- and Asian-Americans | CAM | J Immigr Minor Health | United States | Cross-sectional | **Predictors**:  1-Proportion of individual life in the US  2-Lack of insurance  3- Chronic illness |
|  | Liem and Newcombe., 2021 | Knowledge, attitudes, and usage of complementary-alternative medicine (CAM): A national survey of clinical psychologists in Indonesia | CAM | Current Psychology | Indonesia | Cross-sectional | **Predictors**:  1-Knowledge of CAM  2-Attitudes towards CAM |
|  | Loera et al., 2007 | Predictors of complementary and alternative medicine use among older Mexican Americans | CAM | Complement Ther Clin Pract | United States | Cross-sectional | **Predictors**:  1-Being on Medicaid insurance  2-Frequent church attendance (Religion)  3-Higher number of chronic medical conditions  4-Immigration status  5- Language |
|  | Logiel et al., 2021 | Prevalence and socio-economic factors affecting the use of traditional medicine among adults of Katikekile Subcounty, Moroto District, Uganda | Traditional medicine | African Health Sciences | Uganda | Mix methods | **Predictors**:  1-Long distance to the nearest health center  2-High level of income  3-Positive attitude of the health workers  4-Easy access  5-No cost  **Barriers**:  1- No mechanism has been devised to measure the accurate dosage to be dispensed especially from the herbs used  2-Risk of developing drug-resistant strains of infections |
|  | Matlala et al., 2015 | Confidentiality protection in consulting with modern medicine following use of traditional medicine: perspectives of South African clients | Traditional medicine | Journal of Psychology in Africa | South African | Qualitative study | **Predictors**:  1-Safe  2-Accessible  3-Affordable  4-Culture |
|  | McLaughlin et al., 2012 | Complementary and alternative medicine use among older Australian women--a qualitative analysis | CAM | BMC Complement Altern Med | Australia | Qualitative study | **Predictors**:  1-Influence of practitioner  2-Accessing conventional health services in rural areas  3-Desire to take responsibility for their health and well-being and to make their own healthcare choices  4-Recommendation from friends, family, and other members of the women’s social networks  5-Access to practitioners  **Barriers**:  1-accessing CAM practitioners  2-cost of trip  3-Financial constraints as with other health care providers 4-retaining a rural CAM workforce can also be difficult |
|  | Mhatre et al., 2011 | Influence of benefits, barriers and cues to action for complementary and alternative medicine use among university students | CAM | J Complement Integr Med | Texas | Cross-sectional | **Predictors**:  1-Perceived benefits of CAM usage  2-Native Americans  3-Recommendation by health care provider  4-CAM use by parents and grandparents  **Barrier**:  1-Lack of sufficient scientific testing |
|  | Naja et al., 2015 | The Use of Complementary and Alternative Medicine among Lebanese Adults: Results from a National Survey | CAM | Evid-Based Complement Alternat Med | Lebanon | Cross-sectional | **Predictors**:  1-Belief in the advantages of CAM products  2-Disappointment with conventional medical therapy  3-Physician encouraged the use  4-Increasing income  5-Chronic disease  6-Unmet health need |
|  | Palinkas and Kabongo., 2015 | The use of complementary and alternative medicine by primary care patients. A SURF*NET study | CAM | J Fam Pract | California | Cross-sectional | **Predictors**:  1-Recommendations from friends or coworkers  2-Desire to avoid the side effects of conventional treatments  3-Failure of conventional treatments to cure a problem  ethnicity |
|  | Patterson et al., 2008 | Exploring adolescent complementary/alternative medicine (CAM) use in Canada | CAM | J Interprof Care | Canada | Qualitative study | **Predictors**:  1-Individual influencing factors: Personality traits  2-Culture and media  3-Social contacts within their world  4-Treatment success stories  5-Having knowledge of CAM  6-Supportive of family doctor  7-The essence of the CAM provider’s practice (Their ability to establish a therapeutic relationship)  **Barrier**:  1-Lack of knowledge  2-lack of availability of CAM practices  3-transportation  4-money (Cost)  5-poor advertisement of practices |
|  | Radi et al., 2018 | Attitudes and barriers towards using complementary and alternative medicine among university students in Jordan | CAM | Complementary Therapies in Medicine | Jordan | Cross-sectional | **Barriers**:  1-Doubtful about the effectiveness of CAM.  2- Lack of access to services.  3- Lack of belief in CAM.  4- Lack of knowledge of CAM. |
|  | Rössler et al., 2006 | The use of complementary and alternative medicine in the general population: results from a longitudinal community study | CAM | Psychological Medicine | Switzerland | Longitudinal community study | **Predictors**:  1-Higher physical and psychological complaints |
|  | Roy et al., 2015 | Perception, attitude and usage of complementary and alternative medicine among doctors and patients in a tertiary care hospital in India | CAM | Indian Journal of Pharmacology | India | Cross-sectional | **Predictors**:  1-Positive attitude toward effectiveness, safety, and cost of CAM |
|  | Sarris et al., 2011 | Complementary Medicine Use by Middle-aged and Older Women Personality, Mood and Anxiety Factors | CAM | Journal of Health Psychology | Australia | Longitudinal community study | **Predictors**:  1-More anxiety symptoms.  2-More physical and leisure activities.  3-More consultations with physicians.  4-More health conditions.  5-Openness personality. |
|  | [Sasagawa](https://pubmed.ncbi.nlm.nih.gov/?term=Sasagawa%20M%5BAuthor%5D) et al., 2008 | Positive correlation between the use of complementary and alternative medicine and internal health locus of control | CAM | Explore | United States | Cross-sectional | **Predictors**:  1-High internal health locus of control.  2- Having chronic conditions. |
|  | Schnabel et al., 2014 | Use of complementary and alternative medicine by older adults: a cross-sectional survey | CAM | BMC geriatrics | Germany | Cross-sectional | **Predictors**:  1-Positive preference toward CAM.  2-Statutory insurance.  **Barriers**:  1-Harmful drug interactions.  2-Concern about side effects. |
|  | [Sibbritt](https://www.tandfonline.com/author/Sibbritt%2C+David+W) et al., 2004 | A longitudinal analysis of mid-age women's use of complementary and alternative medicine (CAM) in Australia, 1996-1998 | CAM | Women Health | Australia | Longitudinal study | **Predictors**:  1- Increasing referrals by general practitioners  2-Stopped taking prescription medication.  3-Declined physical health. |
|  | Smith et al., 2008 | Who Is Willing to Use Complementary and Alternative Medicine? | CAM | Explore | United States | Cross-sectional | **Predictors**:  1-Personality characteristics of openness to experience, spirituality, and mood attention. |
|  | Soveri et al., 2020 | Trait reactance and trust in doctors as Predictors of vaccination behavior, vaccine attitudes, and use of complementary and alternative medicine in parents of young children | CAM | PLOS ONE | Finland | Cross-sectional | **Predictors**:  1-Trait reactance |
|  | Thomson et al., 2014 | Psychosocial factors that predict why people use complementary and alternative medicine and continue with its use: a population-based study | CAM | Complementary Therapies in Clinical Practice | Australia | Cross-sectional | **Predictors**:  1-Higher spiritual experience.  2- Higher openness to new experiences.  3- Prescribe by non-medical practitioners. |
|  | Valtonen et al., 2023 | Political orientation predicts the use of conventional and complementary/alternative medicine: A survey study of 19 European countries | CAM | [Social Science & Medicine](https://www.sciencedirect.com/journal/social-science-and-medicine) | Multi country- Europe | Cross-sectional | **Predictors**:  1-Favoring expanded freedoms parties |
|  | Versnik Nowak et al., 2015 | Prevalence and Predictors of Complementary and Alternative Medicine (CAM) Use Among Ivy League College Students: Implications for Student Health Services | CAM | Journal of American College Health | United States | Cross-sectional | **Predictors**:  1-Non-family use of CAM.  2-Positive attitude toward CAM.  3-Health care encounters outcome expectancies.  4-Family use of CAM.  5-Positive personal values outcome expectancies. |
|  | Votova et al., 2006 | Self-care dimensions of complementary and alternative medicine use among older adults | CAM | Gerontology | Canada | Cross-sectional | **Predictors**:  1-Higher spirituality beliefs.  2- Strong self-care attitude.  3-Lower health status. |
|  | Welz et al., 2019 | The importance of herbal medicine use in the German health-care system: prevalence, usage pattern, and influencing factors | Herbal medicine | BMC Health Services Research | Germany | Cross-sectional | **Predictors**:  1-Having chronic diseases.  2-Engaging in physical activities.  3-Going to annual dentist check-ups |
|  | Wheeler et al., 2008 | Dispositional Predictors of complementary medicine and vitamin use in students | CAM | Journal of Health Psychology | United Kingdom | Cross-sectional | **Predictors**:  1-Less rationality trait.  2-Greater absorption trait. |
|  | [Wolsko](https://www.liebertpub.com/doi/abs/10.1089/10755530050120682) et al., 2000 | Alternative/complementary medicine: wider usage than generally appreciated | CAM | The Journal of Alternative and Complementary Medicine | United States | Cross-sectional | **Predictors**:  1-Lower self-rated health status. |
|  | Yeh et al., 2015 | Use of Traditional Medicine and Complementary and Alternative Medicine in Taiwan a Multilevel Analysis | TCIM | Holistic Nursing Practice | Taiwan | Cross-sectional | **Predictors**:  1-Lower health status.  2-Negative experience with Western medical treatment.  3-Lower cost, effect.  4-Higher degree of satisfaction with TM/CAM. |
|  | Zaidi et al., 2022 | Use of complementary and alternative medicine in the general public of Western Saudi Arabia: A cross-sectional survey | CAM | Cureus | Saudi Arabia | Cross-sectional | **Predictors**:  1-Awareness of the services.  2-self-use of the services.  **Barriers**:  1-Lack of trained professionals.  2-Lack of scientific evidence.  3-Long time for treatment.  4-Lack of knowledge about CAM. |
